# Sleep Disordered Breathing in Children with Neuromuscular Disease

**DOI:** 10.3390/children10101675

**Published:** 2023-10-11

**Authors:** Ambika G. Chidambaram, Sanjay Jhawar, Craig M. McDonald, Kiran Nandalike

**Affiliations:** 1 Division of Pediatric Pulmonary and Sleep Medicine, Department of Pediatrics, University of California, Davis, CA 95817, USA; 2 Department of Physical Medicine and Rehabilitation, University of California, Davis, CA 95817, USA

**Keywords:** neuromuscular disease, obstructive sleep apnea, hypoventilation, non-invasive mode of ventilation

## Abstract

Sleep disordered breathing (SDB) in children with neuromuscular disease (NMD) is more prevalent compared to the general population, and often manifests as sleep-related hypoventilation, sleep-related hypoxemia, obstructive sleep apnea, central sleep apnea, and/or disordered control of breathing. Other sleep problems include, sleep fragmentation, abnormal sleep architecture, and nocturnal seizures in certain neuromuscular diseases. The manifestation of sleep disordered breathing in children depends on the extent, type, and progression of neuromuscular weakness, and in some instances, may be the first sign of a neuromuscular weakness leading to diagnosis of an NMD. In-lab diagnostic polysomnography (PSG) remains the gold standard for the diagnosis of sleep disordered breathing in children, but poses several challenges, including access to many children with neuromuscular disease who are non-ambulatory. If SDB is untreated, it can result in significant morbidity and mortality. Hence, we aimed to perform a comprehensive review of the literature of SDB in children with NMD. This review includes pathophysiological changes during sleep, clinical evaluation, diagnosis, challenges in interpreting PSG data using American Academy of Sleep (AASM) diagnostic criteria, management of SDB, and suggests areas for future research.

## 1. Introduction

Sleep disordered breathing (SDB) in children with neuromuscular disease (NMD) is highly prevalent compared to the general population. In a study, Labanowski et al. showed that at least 42% of patients with NMD (only 16% children; with specific NMD diagnoses: myasthenia gravis, myotonic dystrophy, Duchenne muscular dystrophy, inflammatory myopathy) at a NMD clinic in New Mexico (altitude 1500 m) had a respiratory disturbance index of more than 15 [1], which was a higher estimate compared to the general population in the late 1990s. Although this is overall higher than the general population, the prevalence of SDB varies based on the type of NMD and the degree of neuromuscular weakness.

SDB manifests as sleep-related hypoventilation, sleep-related hypoxemia, obstructive sleep apnea (OSA), central sleep apnea (CSA), and/or disordered control of breathing [2]. Other sleep problems include, sleep fragmentation, abnormal sleep architecture, and nocturnal seizures in children with certain neuromuscular diseases [3]. The manifestation of sleep disordered breathing in children depends on the extent, type, and progression of neuromuscular weakness, and in some instances it may be the first sign of a neuromuscular weakness leading to diagnosis of an NMD [4]. The natural history of SDB is variable from one disease to another, and from patient to patient depending on the genotype and phenotype of the NMD [2]. For example, children with DMD often present with SDB such as obstructive sleep apnea, and then develop hypoventilation during sleep and eventually, diurnal respiratory failure as neuromuscular weakness progresses [5].

## 2. Methods

Using PubMed as our primary search engine, relevant articles with the search phrases related to the topic of interest, including, “sleep-disordered breathing in children with neuromuscular disease”, “obstructive sleep apnea and Duchenne muscular dystrophy”, “sleep-disordered breathing and Duchenne muscular dystrophy”, “spinal muscular atrophy and sleep disordered breathing”, “ventilation in children with neuromuscular disease”, “outcomes and ventilatory support in children with neuromuscular disease”.

## 3. Pathophysiology of SDB in Children with NMD

**Figure 1:**
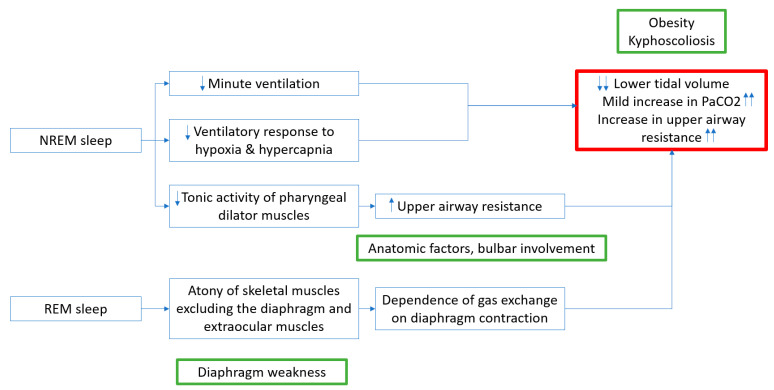
Pathophysiology of sleep in children with NMD. Up arrows indicate increase and down arrows indicate decrease.

In a typically developing child without NMD, during non-rapid eye movement (NREM) sleep, there is decrease in minute ventilation, ventilatory response to hypoxia and hypercapnia, and in the tonic activity of the pharyngeal dilator muscles of the upper airway [3]. The above changes, result in a reduction in tidal volume, an increase in PaCO_2_ (2–6 mmHg from baseline awake PaCO_2_) from awake baseline values, and an increase in upper airway resistance, respectively (Figure 1) [5,6]. Typically developing children without NMD are able to overcome the above changes due to inherent muscular strength, maintaining optimal gas exchange and supporting various body functions during sleep. However, in children with NMD, the above changes are more pronounced, resulting in gas exchange abnormalities, such as, hypoxemia and hypercarbia, due to decreased pulmonary reserve from respiratory muscle weakness [3]. This is also further exacerbated if the patient has a baseline decrease in pulmonary reserve from spine curvature abnormalities, such as kyphosis, due to respiratory muscle weakness, and increased respiratory load from central obesity [3]. Furthermore, children with co-morbid conditions in addition to underlying NMD, such as, macroglossia, adeno-tonsillar hypertrophy, cranio-facial malformations (retrognathia, micrognathia) and crowded oropharynx, are at higher risk of increased upper airway resistance during sleep [7]. Children with bulbar neuromuscular weakness may have difficulty maintaining a potent airway while asleep, and as a result worsening the baseline gas exchange abnormalities during sleep [3].

In rapid eye movement (REM) sleep, we are dependent on the diaphragm for inspiration as there is atonia of the skeletal muscles of respiration (especially, inspiration—external intercostal muscles) [2]. Hence, children with NMD who have diaphragm weakness, are at higher risk for gas exchange abnormalities during REM sleep. Often, the natural history in most children with NMD is due to gas exchange abnormalities in REM sleep first, if diaphragm weakness is predominant, followed by abnormalities in NREM sleep, and eventually to diurnal/awake gas exchange abnormalities [8]. The initial response to hypoxemia and hypercapnia is a higher arousal index, which results in decreased total sleep time, poor sleep quality and sleep fragmentation [5]. This in turn results in daytime fatigue and sleepiness. Over time, with persistent sleep deprivation and sleep fragmentation, the chemoreceptor response to hypoventilation is diminished, and therefore, hypoventilation persists. With progressive weakening of respiratory muscles and diminished chemoreceptor sensitivity to carbon dioxide, daytime hypoventilation ensues [3].

## 4. Clinical Features

Typical symptoms associated with SDB include, snoring, pauses in breathing followed by episodes of gasps for air, frequent awakenings, nocturia, night sweats, and unrefreshed sleep. Some children may also have associated daytime consequences such as, difficulty with attention and concentration. Children, unlike adults, can present with failure to thrive or obesity, due to untreated OSA. Like children who are otherwise healthy, children with NMD can have OSA, due to adenoidal and tonsillar tissue hypertrophy, and therefore, a thorough clinical exam of the visible upper airway is important [7]. Other physical exam findings include, macroglossia relative to the oral cavity, lingual myopathy, crowded oropharynx [3].

Common screening questions for nocturnal hypoventilation in clinical settings include presence of early morning headaches and excessive daytime sleepiness [9]. Excessive daytime sleepiness could be because of poor-quality sleep, due to frequent nighttime awakenings, which in turn could be from physical discomfort, dyspnea, respiratory events (obstructive apneas/hypopneas, central apneas), hypoxemia, hypoventilation and/or spontaneous/non-specific in nature. Often, children with NMD also experience tiredness/fatigue from neuromuscular weakness, and therefore, can be confused and overlapped with a symptom of daytime sleepiness. Hence, differentiation between fatigue and sleepiness is important. Children with hypoventilation also have reported vivid dreams and frequent nightmares [10], and therefore, provide subtle signs to guide further management strategies.

## 5. SDB, Specific to Type of NMD

As highlighted above, the extent of SDB and hypoventilation is dependent on the type and progression of NMD. In children with muscular weakness, such as, Duchenne muscular dystrophy, a bi-modal distribution is seen. In the earlier age group where respiratory muscle function is preserved, they tend to present with OSA which is further exacerbated by excess weight gain, which is a therapeutic side effect from chronic steroid therapy. In the older children, as respiratory muscle weakness worsens, then, hypoventilation ensues [11]. On the other hand, children with myotonic dystrophy type 1 exhibit daytime sleepiness that is likely multifactorial: untreated SDB, elevated periodic limb movement index, central alveolar hypoventilation, and potentially a central cause for daytime sleepiness. The SDB described in children with myotonic dystrophy type 1 is typically a combination of obstructive sleep apnea and central sleep apnea [12,13]. In adults with neuromuscular weakness and severe cardiomyopathy with heart failure, a central type of sleep disordered breathing pattern has been reported but not commonly in children with cardiomyopathy [4].

Children with a neuro-muscular junction abnormalities, such as myasthenia gravis, have been reported to have OSA and central sleep apnea (CSA), due to upper airway muscular weakness and can progress to fulminant respiratory failure if not treated [2].

Children with a peripheral neuropathy, such as, Charcot Marie Tooth disease, are at higher risk of SDB, primarily OSA, suggested to be due to phrenic and pharyngeal neuropathy. The exact mechanism for SDB is unclear [2].

In children with lower motor neuronopathies, such as, spinal muscular atrophy (SMA), SDB has been reported. Chacko et al., performed a prospective cohort study in children with SMA type 1 to 3 under the age of 19 years and described the PSG findings [14]. They reported an increase in respiratory events in REM sleep compared to NREM sleep across all types of SMA, and this is likely due to the weakness in the skeletal muscles of respiration, external and internal intercostal muscles. Both central and obstructive events (central > obstructive) were more common in SMA type 1. Mixed sleep apnea was seen in SMA types 1 and 2 [14].

Children with upper motor neuronopathies, typically due to hypoxic encephalopathy, are at higher risk of SDB, due to abnormal tone of the upper airway muscles and decreased pulmonary reserve due to impaired airway clearance and weakness of the respiratory muscles to support the thoracic cage [2].

In children with brainstem abnormalities, such as, Arnold Chiari malformation and myelomeningocele, the respiratory drive may be affected as the respiratory control centers are located in the brainstem. Therefore, they tend to have a predominance of central events compared to obstructive events. Lastly, children with spinal cord injuries, are also at high risk of SDB depending on the level of spinal cord involvement (incomplete or complete) [2]. Both, OSA and CSA, are highly prevalent in patients with spinal cord injuries, and severity is higher in those with complete spinal cord injuries [15,16]. Those with cervical cord injuries, specifically, above C4, have weakness of the diaphragm resulting in respiratory insufficiency while awake as well [15].

## 6. Evaluation of SDB in Children with NMD

The clinical features of SDB in children with NMD are not specific to a particular SDB, and children with SDB can be asymptomatic during the daytime [11]. There are also no questionnaires currently available that are validated for children with NMD to assess for SDB. However, sleep quality has been assessed in a natural history study using the Pittsburg Sleep Quality Index (PSQI) in children with DMD that was led by the Cooperative International Neuromuscular research group. They showed that poor sleep quality assessed using PSQI independently correlated with decreased FVC and increased body weight, however correlation with presence and severity of SDB was not assessed [17]. Therefore, an objective evaluation by performing a polysomnography (PSG) in a pediatric sleep laboratory to further characterize the type and severity of SDB, and abnormalities during sleep, is required [18]. Although obtaining a PSG in real-time would be ideal, it is often not possible as there are several challenges, therefore, surrogate tests are in demand to diagnose and characterize SDB in children with NMD. However, there has not been a surrogate test that has shown to accurately diagnose SDB. Some of the surrogate tests commonly used, include, pulmonary function test (PFT), nocturnal oximetry and nocturnal capnography monitoring [18].

### 6.1. Pulmonary Function Tests (PFT)

PFTs are often readily available and accessible in most NMD centers. Frohlich et al., performed a retrospective chart review on 50 children with progressive NMD in a single center tertiary care children’s hospital in Australia [19]. They found a statistically significant association between hypoventilation during sleep and FVC less than 60% predicted (using Global Lung Initiative reference equations) and a z-score less than −3.24. The sensitivity was 78% and specificity was 73% for FVC < 60% predicted or z-score < −3.24 to predict presence of hypoventilation during sleep. They also demonstrated scoliosis as a factor that predicted hypoventilation during sleep. Similar results were reported in another cohort of children with progressive NMD in Canada [20]. In addition, they showed an increase in residual volume and total lung capacity and a decrease in respiratory muscle function after a year following onset of hypoventilation during sleep [20]. PFT parameters currently serve as tests that help with counseling parents and children with NMD to obtain a PSG to screen for SDB, including sleep related hypoventilation [21].

### 6.2. Overnight Oximetry

**Figure 2:**
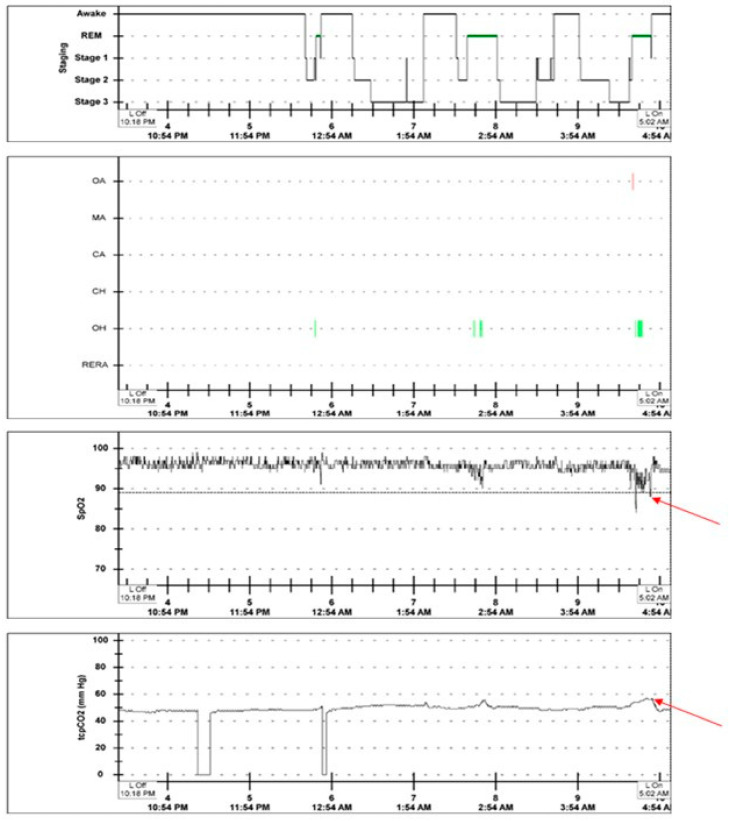
Sleep stages, respiratory events, pulse oximetry and transcutaneous capnography data taken from the hypnogram of a 17-year old teenager with Duchenne muscular dystrophy who underwent an overnight in-laboratory polysomnogram in room air. The above figure illustrates the hypnogram, consisting of sleep stages, respiratory events, trends of pulse oximetry and transcutaneous capnography data of a 17-year old teenager with Duchenne muscular dystrophy who underwent an overnight in-laboratory polysomnogram in room air. The red arrows indicate sustained desaturations (lowest SpO_2_ was 84%) and CO_2_ retention during REM sleep. There were obstructive hypopneas (OH indicated as the green vertical line) and one obstructive apnea (OA indicated as the red vertical line) that were associated with oxyhemoglobin desaturations and hypercapnia. Abbreviations: REM—rapid eye movement, OA—obstructive apnea, MA—mixed apnea, CA—central apnea, CH—central hypopnea, OH—obstructive hypopnea, RERA—respiratory effort related arousal, SpO_2_—Oxyhemoglobin saturation, tcpCO_2_—transcutaneous carbon dioxide, mmHg—millimeters of Mercury, L Off- lights off, L On—lights on.

Overnight oximetry is used as a surrogate diagnostic tool for SDB [18]. The diagnosis is based on the pattern of oxyhemoglobin desaturations and the duration of time spent with SpO_2_ ≤ 90% in children [22]. Typically, if OSA is the predominant clinical process, then a ‘saw-toothed’ pattern will be seen with the desaturations, as most desaturations will return to baseline following an apneic or hypopneic event. If hypoventilation is the predominant clinical process, then a more sustained desaturation is typically seen (Figure 2) [23]. However, hypoventilation can continue to exist despite normal oxyhemoglobin saturations, hence, emphasizing the importance of capnography. Other limitations include, variability in signal averaging times and artefacts related to poor perfusion, and/or movement [3].

### 6.3. Capnography

Sleep-related hypoventilation has been defined by the “American Academy of Sleep Medicine Manual for the Scoring of Sleep and Associated Events” released in 2007, as PCO_2_ (arterial PCO_2_ or surrogate marker) above 50 mmHg for more than 25% of total sleep time [24]. In clinical practice, we use transcutaneous or end-tidal CO_2_ monitoring as part of a polysomnography to provide the above measure. However, due to the difficulties in obtaining an in-laboratory polysomnography, ambulatory transcutaneous capnography has been studied as a surrogate diagnostic test to evaluate for sleep related hypoventilation [25,26,27].

Felemban et al. and Griffon et al. showed ambulatory transcutaneous capnography was feasible but did not study diagnostic accuracy for sleep related hypoventilation [26,27]. Shi et al., on the other hand, showed that ambulatory transcutaneous capnography had poor diagnostic accuracy compared to end-tidal CO_2_ or transcutaneous CO_2_ monitoring performed by an in-laboratory polysomnography; however, all families preferred ambulatory monitoring compared to in-laboratory evaluation [25]. Hence, further studies are required to improve diagnostic accuracy of ambulatory transcutaneous capnography as this would increase access to reliable diagnostic modalities for sleep related hypoventilation in children with NMD.

### 6.4. Polysomnography

PSG with capnography performed in a sleep laboratory is considered and remains the gold standard in the diagnosis of SDB in children with neuromuscular disease [18]. In addition to providing a diagnosis, a polysomnography also aids in providing information to aid clinicians to initiate the appropriate respiratory support [28]. This is especially important in considering initiation of respiratory support in a child who is unable to perform pulmonary function tests.

In most pediatric sleep centers, PSG provides information regarding sleep stages, sleep fragmentation, obstructive apnea hypopnea index, central apnea index, respiratory rate, periodic limb movement index, single lead EKG data, SpO_2_ data, end-tidal CO_2_/transcutaneous CO_2_, and presence/absence of abnormal behavior during sleep [24]. The emphasis on capnography, either measured as end-tidal carbon dioxide or/and transcutaneous carbon dioxide, is very important in formulating an appropriate treatment plan regarding choice of respiratory support [18]. The sleep study is then reviewed and scored based on the criteria published on the “American Academy of Sleep Medicine Manual for the Scoring of Sleep and Associated Events” [24]. One of the challenges in scoring respiratory events in children with neuromuscular weakness is the lack of thoraco-abdominal paradox due to muscle weakness during an obstructive event and therefore, can mimic a central event [5].

Despite valuable information obtained using an overnight PSG, there are several challenges and limitations regarding access to in-laboratory polysomnography [18]. To state a few: limited access to pediatric sleep laboratories which can be more challenging to a child with NMD who is non-ambulatory; high wait times to see a pediatric sleep and/or pulmonary provider; patient’s sleep at a laboratory may not be representative of their sleep at home, inability to achieve REM sleep, inability to fall asleep in an environment outside home, inability to tolerate the various PSG sensors/monitors; inability to fall asleep due to co-existing sleep disorders such as, insomnia, circadian rhythm disorders [29,30]. An ambulatory polysomnography with capnography would be ideal, but currently not clinically validated for use in children [29]. Reassuringly, there are a few promising groups studying the utility of ambulatory polysomnography, which would be clinically useful and helpful to patients with NMD in the future.

## 7. Management

### 7.1. Non-Invasive Ventilatory (NIV) Support

Depending on the type and severity of SDB and severity of hypoventilation (if present), non-invasive ventilatory (NIV) support can be initiated as continuous positive airway pressure (CPAP), bilevel positive airway pressure (BPAP) support, or volume ventilator support [31]. If there is clinical evidence of respiratory muscle weakness and/or hypoventilation, then initiation of BPAP with a back-up rate or use of a volume ventilator support is recommended, to optimize ventilation. CPAP therapy alone is considered inappropriate in children with NMD and respiratory muscle weakness, as it does not support ventilation and may further stress respiratory muscles worsening fatigue.

In addition to providing the optimal NIV settings; selection of the right mask and fit is also important [31]. A nasal mask is recommended instead of a full-face mask, as there is higher risk of aspiration due to inability of the child with neuromuscular weakness to remove the full-face mask in the event of vomiting. Despite a need to find the optimal mask fit, there are very few models available for the pediatric population, therefore finding an appropriate mask is often challenging. There is also a risk for facial deformations such as, mid-face hypoplasia with use of nasal masks, especially in children when NIV is initiated in the pre-pubertal years. Other side effects from long term NIV use include: dryness in the eyes, nose or mouth, skin rash or irritation, nasal congestion, sinus infections, and/or aerophagia especially with BPAP use [32]. Continued follow-up after initiation of NIV can help screen for commonly seen side effects and treat them in a timely manner. In children with NMD, who also have diurnal hypoventilation, then NIV can still be used to provide ventilatory support continuously [3]. Depending on the patient, NIV support can be given in the form of mouthpiece ventilation during the daytime, and at night, without using tracheostomy [3,31]. However, swallowing and secretions control require to be preserved while initiating mouthpiece ventilation. If not, NIV can still be provided continuously via nasal mask [33].

There are no established clinical criteria for initiation of NIV support, however, specific NMD groups have their own clinical guidelines. For example, the indications for initiation of non-invasive support for a child with DMD include end-tidal carbon dioxide (ETCO_2_) or transcutaneous carbon dioxide (TCPCO_2_) 50 mmHg or more for at least 2% of total sleep time, 10 mmHg or higher increase of end-tidal carbon dioxide or transcutaneous carbon dioxide 10 mmHg from awake baseline, if SpO_2_ 88% or lower for at least 2% of total sleep time or ≥5 min continuously, or an apnea-hypopnea index of ≥5 events/hour [22]. Another group of conference participants recommended initiation of NIV when there are symptoms of abnormal gas exchange and one of the following: PCO_2_ ≥ 45 mmHg, SpO_2_ ≤ 88% for >5 min continuously during sleep, or maximal inspiratory pressure < 60 cm of water or forced vital capacity (FVC) < 50% predicted [21]. There are recommendations provided based on data obtained from queries to experts in the field of NMD and the respiratory system, as randomized controlled trials are difficult to perform due to a variety of reasons, most importantly including, ethical considerations, and low sample size.

Despite presence of objective indicators to initiate NIV, initiation of NIV too early or before onset of symptoms, can result in poor adherence to NIV [33]. Moreover, children with NMD who are non-ambulatory, often do not have a daily/bedtime routine that they follow, and as result have difficulty including NIV in their routine unless clinically symptomatic. Hence, initiation of NIV with onset of nighttime/daytime symptoms in addition to the presence of gas exchange abnormalities during sleep is considered to be reasonable time to initiate NIV support [3]. A randomized controlled trial performed by Ward et al. in children and adults with congenital NMD or those with chest wall disease and vital capacity that was less than 50% predicted who had nocturnal hypoventilation and daytime normocapnia, were randomized to a group that received NIV and to a control group that did not [34]. However, there was a third group that was created to support patients with NIV in the control group if they developed any one of the following: worsening nighttime symptoms, daytime hypercapnia, recurrent respiratory tract infections (>3 episodes per year), poor growth resulting in failure to thrive and acute respiratory failure resulting in a hospitalization. All study participants were followed for 24 months, and only one patient in the control group did not need NIV support at the 24-month follow-up, and this patient had congenital scoliosis and not a NMD per se, which may have delayed need for NIV initiation. About 70% of patients in the control group who completed the study, required initiation of NIV within 12 months of diagnosis of nocturnal hypoventilation. However, two of the patients refused NIV support and developed respiratory failure within 12 months. Hence, this study showed that diurnal hypercapnic respiratory failure ensued within 12–24 months of onset of nocturnal hypoventilation. This can used as a point of discussion with patients of NMD and their family members when considering initiation of respiratory support as their disease progresses.

Once NIV has been initiated, a titration PSG or close bedside monitoring is recommended to guide optimal settings [18]. Since an intrinsic lung disease is not expected to be present in a child with NMD, the goal is to titrate settings to achieve an ETCO_2_ or TCPCO_2_ between 35 and 45 mmHg and SpO_2_ ≥ 95% without use of supplemental oxygen [3]. Although the main goals are to optimize gas exchange abnormalities, decrease cardiac afterload, correct SDB and normalize sleep architecture, the goal to improve overall quality of life, cannot be more emphasized. This was further elucidated by a qualitative study performed in children with DMD, who had used respiratory support (mainly iron lung) to support breathing during sleep [35]. They reported that most caregivers chose the respiratory support as more of a comfort measure rather than in the hopes of extending life as most NMDs are progressive or irreversible. This, however, will need to be re-evaluated as more disease modifying therapies are now available, and many are being studied. Hence, an informed discussion with families regarding long term ventilation is very important before initiating NIV support in children with NMD.

Several studies have shown a positive impact of using NIV on the clinical outcomes in children with NMD. A retrospective review of 835 patients with DMD at a NMD center in Naples, Italy, performed by Passamano et al. [36], revealed a statistically significant improvement in the survival rate among DMD patients born between 1981 and 1990, compared to the patients born between the 1961 and 1970 cohort, and between the 1971 and 1980 cohort. This was attributed to improved airway clearance techniques, introduction of NIV, and cardiomyopathy management. The average age for respiratory related deaths was significantly lower in the NIV group (average age 27.9 years; range 23 to 38.6 years of age) compared to the non-NIV group, which was 17.7 years (range 11.6 to 27.5 years) in the non-NIV group. A recent systematic review and meta-analysis on the outcomes of children (0 to 18 years of age) with NMD using long-term NIV support [37], showed a decrease in mortality compared to supportive care, except in children with SMA type 1, where invasive mode of ventilation had decreased mortality compared to NIV. However, children with SMA type 1 who received nusinersen, had a similar mortality rate for the NIV and invasive mode of ventilation groups. Katz et al. also demonstrated a decrease in the hospitalizations with initiation of NIV compared to pre-NIV initiation as the participants served as their own control. The study also showed improvement in the AHI, TC PCO_2_ levels and desaturation episodes [38]. Similar trends were seen in the cohort analyzed by Dohna-Schwake et al. from Germany, including a decrease in the number of respiratory tract infections, including the ones needing antibiotic use, and also an overall decrease in the hospitalization rates [39]. In a cohort of children with SMA type 1, the investigators reported to be able to prevent pectus excavatum by using positive inspiratory pressures and positive end-expiratory pressures, which is promising [40].

With more and more disease modifying drugs being available for pediatric NMDs, this may reverse the disease process and improve respiratory muscle strength. Hence, recently, Gurbani et al., have postulated NIV and invasive ventilatory support weaning strategies in the children with improvement in respiratory muscle strength [28]. This is a great accomplishment for both the children and families with NMD and researchers in this field.

Although NIV has proven to show positive outcomes in children with NMD, adherence to NIV remains an issue. Hurvitz and Sunkonkit et al. [41], have shown through a retrospective analysis of data collected from three major centers caring for fifty-nine children with DMD and on NIV. They found that those who were older, had a lower FVC, and those who were not on deflazacort were associated with adherence to NIV, but were not statistically significant when included in the multivariable analysis. Hence, further studies are required to study factors that influence adherence to NIV.

### 7.2. Ventilatory Support via Tracheostomy

In those children with NMD who are unable to successfully adapt to NIV due to various reasons: poor mask fit, inability to tolerate pressures, very poor NIV adherence in the setting of gas exchange abnormalities while awake, patient preference, inability to control oral secretions with very high risk of aspiration, tracheostomy can be used to provide ventilatory support maybe considered [31]. Tracheostomy placement requires additional caregiver training, which can take several weeks of inpatient hospital stay to accomplish. The patients also became less independent and more dependent on caregivers for routine respiratory care, including airway clearance. Phonation also becomes challenging but can be established in some patients with a speaking valve [31]. The patients are also at higher risk for respiratory arrest due to mucus plugging.

## 8. Conclusions and Future Research

SDB is highly prevalent among children with NMD, and therefore utilizing appropriate screening measures and initiating appropriate respiratory support are important to improve outcomes and quality of life. As more disease modifying therapies are becoming more available and accessible, the natural history of children with NMD is likely to change and therefore, modifications to existing clinical guidelines would be necessary. Some children with progressive NMD still do not have disease modifying therapies, and therefore, continued surveillance and early discussion of goals of care continue to remain necessary. There is also a need for advancements of precision medicine in this field to help tailor to the needs of every patient as the natural history, severity of muscular weakness, and resultant disability are variable from one NMD to another, and also variable from one patient to another.

## Data Availability

Not applicable.

## References

[1] Labanowski M., Schmidt-Nowara W., Guilleminault C. (1996). Sleep and neuromuscular disease: Frequency of sleep-disordered breathing in a neuromuscular disease clinic population. Neurology.

[2] Arens R., Muzumdar H. (2010). Sleep, sleep disordered breathing, and nocturnal hypoventilation in children with neuromuscular diseases. Paediatr. Respir. Rev..

[3] Panitch H.B. (2017). Respiratory Implications of Pediatric Neuromuscular Disease. Respir. Care.

[4] Aboussouan L.S. (2015). Sleep-disordered Breathing in Neuromuscular Disease. Am. J. Respir. Crit. Care Med..

[5] Sawnani H. (2019). Sleep disordered breathing in Duchenne muscular dystrophy. Paediatr. Respir. Rev..

[6] Henke K.G., Badr M.S., Skatrud J.B., Dempsey J.A. (1992). Load compensation and respiratory muscle function during sleep. J. Appl. Physiol..

[7] Katz E.S., D’Ambrosio C.M. (2010). Pediatric obstructive sleep apnea syndrome. Clin. Chest Med..

[8] Fauroux B., Khirani S. (2014). Neuromuscular disease and respiratory physiology in children: Putting lung function into perspective. Respirology.

[9] Wang D., Piper A.J., Yee B.J., Wong K.K., Kim J.W., D’Rozario A., Rowsell L., Dijk D.-J., Grunstein R.R. (2014). Hypercapnia is a key correlate of EEG activation and daytime sleepiness in hypercapnic sleep disordered breathing patients. J. Clin. Sleep. Med..

[10] Bourke S.C. (2014). Respiratory involvement in neuromuscular disease. Clin Med..

[11] Suresh S., Wales P., Dakin C., Harris M.A., Cooper D.G. (2005). Sleep-related breathing disorder in Duchenne muscular dystrophy: Disease spectrum in the paediatric population. J. Paediatr. Child. Health.

[12] Quera Salva M.A., Blumen M., Jacquette A., Durand M.C., Andre S., De Villiers M., Eymard B., Lofaso F., Heron D. (2006). Sleep disorders in childhood-onset myotonic dystrophy type 1. Neuromuscul. Disord..

[13] Ho G., Cardamone M., Farrar M. (2015). Congenital and childhood myotonic dystrophy: Current aspects of disease and future directions. World J. Clin. Pediatr..

[14] Chacko A., Sly P.D., Gauld L. (2020). Polysomnography findings in pediatric spinal muscular atrophy types 1–3. Sleep Med..

[15] Chiodo A.E., Sitrin R.G., Bauman K.A. (2016). Sleep disordered breathing in spinal cord injury: A systematic review. J. Spinal Cord. Med..

[16] Sankari A., Bascom A., Oomman S., Badr M.S. (2014). Sleep disordered breathing in chronic spinal cord injury. J. Clin. Sleep Med..

[17] Newitt J., Gordish-Dressman H., Clemens P., Strollo P., Investigators C.D. (2019). Identifying Potential Predictive Factors of Sleep Quality in Duchenne Muscular Dystrophy. Am. J. Respir. Crit. Care Med..

[18] Katz S.L. (2009). Assessment of sleep-disordered breathing in pediatric neuromuscular diseases. Pediatrics.

[19] Frohlich M., Widger J., Thambipillay G., Teng A., Farrar M., Chuang S. (2022). Daytime predictors of nocturnal hypercapnic hypoventilation in children with neuromuscular disorders. Pediatr. Pulmonol..

[20] Katz S.L., Gaboury I., Keilty K., Banwell B., Vajsar J., Anderson P., Ni A., MacLusky I. (2010). Nocturnal hypoventilation: Predictors and outcomes in childhood progressive neuromuscular disease. Arch. Dis. Child..

[21] Finder J.D., Birnkrant D., Carl J., Farber H.J., Gozal D., Iannaccone S.T., Kovesi T., Kravitz R.M., Panitch H., Schramm C. (2004). Respiratory care of the patient with Duchenne muscular dystrophy: ATS consensus statement. Am. J. Respir. Crit. Care Med..

[22] Birnkrant D.J., Bushby K., Bann C.M., Alman B.A., Apkon S.D., Blackwell A., Case L.E., Cripe L., Hadjiyannakis S., Olson A.K. (2018). Diagnosis and management of Duchenne muscular dystrophy, part 2: Respiratory, cardiac, bone health, and orthopaedic management. Lancet Neurol..

[23] Perrin C., Unterborn J.N., Ambrosio C.D., Hill N.S. (2004). Pulmonary complications of chronic neuromuscular diseases and their management. Muscle Nerve.

[24] Berry R.B., Budhiraja R., Gottlieb D.J., Gozal D., Iber C., Kapur V.K., Marcus C.L., Mehra R., Parthasarathy S., Quan S.F. (2012). Rules for scoring respiratory events in sleep: Update of the 2007 AASM Manual for the Scoring of Sleep and Associated Events. Deliberations of the Sleep Apnea Definitions Task Force of the American Academy of Sleep Medicine. J. Clin. Sleep Med..

[25] Shi J., Chiang J., Ambreen M., Snow N., Mocanu C., McAdam L., Goldstein R.S., Rose L., Amin R. (2023). Ambulatory transcutaneous carbon dioxide monitoring for children with neuromuscular disease. Sleep Med..

[26] Griffon L., Touil S., Frapin A., Khirani S., Amaddeo A., Fauroux B. (2018). Home monitoring of transcutaneous PCO_2_ in children on long term ventilation. Eur. Respir. J..

[27] Felemban O., Leroux K., Aubertin G., Miandy F., Damagnez F., Amorim B., Ramirez A., Fauroux B. (2011). Value of gas exchange recording at home in children receiving non-invasive ventilation. Pediatr. Pulmonol..

[28] Gurbani N., Pascoe J.E., Katz S., Sawnani H. (2021). Sleep disordered breathing: Assessment and therapy in the age of emerging neuromuscular therapies. Pediatr. Pulmonol..

[29] Marcus C.L., Traylor J., Biggs S.N., Roberts R.S., Nixon G.M., Narang I., Bhattacharjee R., Davey M.J., Horne R.S., Cheshire M. (2014). Feasibility of comprehensive, unattended ambulatory polysomnography in school-aged children. J. Clin. Sleep Med..

[30] Green A., Nagel N., Kemer L., Dagan Y. (2022). Comparing in-lab full polysomnography for diagnosing sleep apnea in children to home sleep apnea tests (HSAT) with an online video attending technician. Sleep. Biol. Rhythms.

[31] Alexiou S., Piccione J. (2017). Neuromuscular disorders and chronic ventilation. Semin. Fetal Neonatal Med..

[32] Carron M., Freo U., BaHammam A.S., Dellweg D., Guarracino F., Cosentini R., Feltracco P., Vianello A., Ori C., Esquinas A. (2013). Complications of non-invasive ventilation techniques: A comprehensive qualitative review of randomized trials. Br. J. Anaesth..

[33] Panitch H.B. (2010). Diurnal hypercapnia in patients with neuromuscular disease. Paediatr. Respir. Rev..

[34] Ward S., Chatwin M., Heather S., Simonds A.K. (2005). Randomised controlled trial of non-invasive ventilation (NIV) for nocturnal hypoventilation in neuromuscular and chest wall disease patients with daytime normocapnia. Thorax.

[35] Miller J.R., Colbert A.P., Schock N.C. (1988). Ventilator use in progressive neuromuscular disease: Impact on patients and their families. Dev. Med. Child. Neurol..

[36] Passamano L., Taglia A., Palladino A., Viggiano E., D’Ambrosio P., Scutifero M., Rosaria Cecio M., Torre V., De Luca F., Picillo E. (2012). Improvement of survival in Duchenne Muscular Dystrophy: Retrospective analysis of 835 patients. Acta Myol..

[37] AlBalawi M.M., Castro-Codesal M., Featherstone R., Sebastianski M., Vandermeer B., Alkhaledi B., Bedi P.K., Abusido T., MacLean J.E. (2022). Outcomes of Long-Term Noninvasive Ventilation Use in Children with Neuromuscular Disease: Systematic Review and Meta-Analysis. Ann. Am. Thorac. Soc..

[38] Katz S., Selvadurai H., Keilty K., Mitchell M., MacLusky I. (2004). Outcome of non-invasive positive pressure ventilation in paediatric neuromuscular disease. Arch. Dis. Child..

[39] Dohna-Schwake C., Podlewski P., Voit T., Mellies U. (2008). Non-invasive ventilation reduces respiratory tract infections in children with neuromuscular disorders. Pediatr. Pulmonol..

[40] Bach J.R., Bianchi C. (2003). Prevention of pectus excavatum for children with spinal muscular atrophy type 1. Am. J. Phys. Med. Rehabil..

[41] Hurvitz M., Sunkonkit K., Defante A., Lesser D., Skalsky A., Orr J., Chakraborty A., Amin R., Bhattacharjee R. (2023). Non-invasive ventilation usage and adherence in children and adults with Duchenne muscular dystrophy: A multicenter analysis. Muscle Nerve.

